# Impact of ideal versus estimated body weight on haemofiltration dosing in critically ill patients with AKI

**DOI:** 10.1186/cc13594

**Published:** 2014-03-17

**Authors:** F Fischer, A Putignano, C Willars, J Wendon, S Harris, G Auzinger

**Affiliations:** 1 Kings College Hospital, London, UK

## Introduction

Acute kidney injury (AKI) in the critically ill is an independent risk factor for adverse outcome [1]. Previously, it was suggested that high-volume haemofiltration (HVHF) may confer a mortality benefit and lead to a reduction in organ failure compared with standard ultrafiltration rates (UF) [2]. This was not confirmed by a recent investigation [3]. It has also not been determined whether ideal (IBW) or estimated/actual body weight (E/ABW) was used to calculate UF rates. We sought to determine what impact different weight calculations have on delivered UF rates.

## Methods

A retrospective single-centre study in a tertiary referral institution. Continuous venovenous haemodiafiltration (CVVHDF) was administered according to the patient's IBW. The delivered UF rate was then calculated both for IBW and E/ABW. The latter was based on measurements obtained at the time of ICU admission. We compared the highest predilution, postdilution and dialysate volume administered each day according to the different weight estimate measurements respectively. Student's *t *tests and chi-square tests were used for statistical analysis (*P *< 0.05).

## Results

Data from 33 patients receiving renal replacement therapy were analysed. Mean time (± SD) of treatment interruption was 1.4 ± 2.7 hours/day due to filter changes, transfers for CT scans and surgical procedures. Therefore, 94% of the prescribed dose was delivered. There was a mean of 2.3 filtration dose prescriptions per patient over the observation period (due to changing clinical conditions), with a total of 77 dose adaptations. Mean E/ABW was significantly higher than calculated IBW (76.3 kg vs. 61.4 kg), and thus a difference of 14.9 kg (95% CI = 9.2 to 20.6 kg). This resulted in a significant difference in mean filtration dose delivered of 33.1 ml/kg/ hour for E/ABW versus 39.7 ml/kg/hour for IBW respectively (*P *< 0.001). As a consequence, in 29.6% (8/27) of cases where HVHDF (>35 ml/kg/ hour) was prescribed, standard volume haemodiafiltration (SVHDF) (≤35 ml/kg/hour) was delivered. In 10% (5/50) of cases where SVHDF was prescribed, HVHDF was delivered (*P *< 0.001).

## Conclusion

We conclude that the delivered UF rate in our cohort of patients differed significantly depending on measured or calculated body weight. In almost one-third of cases where HVHDF was prescribed, SVHDF was delivered. As many interventions in the ICU are based on IBW and daily weighing of patients is not uniformly practiced, this makes comparison of data difficult.
